# Circulatory Support as a Bridge to Pediatric Heart Transplantation

**DOI:** 10.5935/abc.20160047

**Published:** 2016-04

**Authors:** Fernando A . Atik

**Affiliations:** Instituto de Cardiologia do Distrito Federal, Brasília, DF - Brazil

**Keywords:** Advanced Cardiac Life Support, Heart Defects, Congenital, Heart Transplantation, Outcome Assessment (Health Care)

Dear Editor,

The article "Use of short-term circulatory support as a bridge in pediatric transplantation", recently published^1^ in *Arquivos Brasileiros de Cardiologia* has aroused great interest.

Caneo et al.^1^ published the largest national experience with the use of circulatory support in children. The authors, according to the reported experience, demonstrated that the use of ventricular assist devices increased the possibility for children in cardiogenic shock to undergo transplantation, although mortality outcomes remained very high, according to international experiences.^2,3^

Although it is a noteworthy experience for Brazil, it is appropriate that some details should be observed.

About the risk stratification, for instance, Caneo et al.^1^ grouped patients in Interagency Registry for Mechanically Assisted Circulatory Support (INTERMACS) 1 and 2 in the same category, which certainly results in differences in shock severity and therapeutic response.

More important than the initial assessment is the patient's response to treatment. It is absolutely necessary that the child have time for correction of multiple-organ dysfunction prior to the transplantation. Caneo et al.^1^ had a mean time of 19 days to perform the transplantation in the group undergoing mechanical circulatory assistance, but one patient was transplanted within 6 hours! How do the authors manage the assisted child in relation to maintenance or not in the transplant waiting list? What are the recipient's minimum conditions to accept a possible donor during this circulatory assistance phase?

Resource allocation is limited in our country, so it is important to use them sensibly and in those with a better chance of survival. Additionally, the number of donors is insufficient to meet the demand of recipients. Wouldn't the use of a donor to a recipient in INTERMACS 1 and 2 be a waste of a donor to another recipient with better chances? Ethical dilemmas are certainly involved in this discussion.

I would like to congratulate Caneo et al.^1^ for bringing such an important experience into the Brazilian cardiology community. Last but not least, the lack of availability of this technology in our country constitutes a serious problem, which must have the support of the competent entities, so that there is training and rationalization of use in heart transplantation reference centers.
