# Supplementary material for: Circulatory Support as a Bridge to Pediatric Heart Transplantation
Source: Arq Bras Cardiol. 2016 Apr;106(4):350–1. doi: 10.5935/abc.20160047 (PMC4845712; doi:10.5935/abc.20160047)
Supplement: Related texts

# Reply

**Authors:** Luiz Fernando Caneo, Leonardo A. Miana, Marcelo B. Jatene

Dear Editor,

The article "Use of short-term circulatory support as a bridge in pediatric transplantation" represents our initial experience with short-term circulatory assist devices, more precisely the use of both extracorporeal membrane oxygenation (ECMO) and the centrifugal pump for this purpose. The concepts and clinical management in this phase, explained in the manuscript, represented an important step in the development of our team. Although our mechanical circulatory support (MCS) program was started in 1999, only recently, after adequate investment in equipment and training of our staff, we achieved more favorable results with ECMO.^1^

Our recently published experience with pediatric heart transplantation (HTx)^2^ shows that, until April 2012, we performed 114 HTx and used ECMO in only two patients. Over the past three years, however, there has been an exponential increase in that number and we performed more than 70 HTx using more than 25 MCS devices (unpublished data). Certainly, the use of MCS has contributed to this increase in volume, due the inclusion of borderline recipients, Interagency Registry for Mechanically Assisted Circulatory Support (INTERMACS) 1 and 2, not so well compensated clinically. Moreover, we observed a 70% increase in the number of congenital heart disease as a cause of HTx indication in our list. Our early mortality, which was consistent with the international literature,^1^ increased as a result of the practice described above,^2^ which made us review our protocols. Many of the questions raised in the letter sent by Atik had already been disscussed in our team.

It is known that short-term MCS devices was not designed to be used as a bridge to the HTx, and this is well emphasized in our manuscript, as well as the inherent characteristics of the Brazilian public health service, in which we do not have access to more appropriate devices. On the other hand, the mortality of these patients in the waiting list, according to our experience and before the advent of the MCS, was markedly elevated,^3^ which led us to invest in the program.

Among the lessons learned, it is now known that patients that have some dysfunction in other organs, in addition to heart, are removed from the waiting list until the problem resolved.

The mentioned case that remained in the waiting list for a very short time refers to an acute failure in a patient already listed, who, after the setting up of the MCS, received an organ, in fact, is only an anecdotal case.

Currently, our service has launched the clinical protocol of the pneumatic paracorporeal ventricular assist device developed in our Department of Bioengineering, which is available in sizes 15, 30 and 65 mL for institutional patients. We live in a time of greater maturity in clinical management, anticoagulation and nutritional support with the use of these devices.

Regarding the ethical aspects mentioned in the letter, we would like to emphasize that the proper allocation of organs to these patients is a constant concern of our team. To consider the use of an borderline donor in unfavorable cases may be a viable alternative. By proposing the use of MCS to the family, we must keep in mind that we are not necessarily heading towards HTx and that will only be considered at the appropriate time. To recognize our limitations is part of the evolution of a high-quality MCS and HTx program.

**Sincerely,** Luiz Fernando Caneo Leonardo A.
Miana Marcelo B. Jatene

## References

[1] Miana LA, Caneo LF, Tanamati C, Penha JG, Guimarães VA, Miura N (2015). Post-cardiotomy ECMO in pediatric and congenital heart
surgery: impact of team training and equipment in the
results. Rev Bras Cir Cardiovasc.

[2] Miana LA, Azeka E, Caneo LF, Turquetto AL, Tanamati C, Penha JG (2014). Pediatric and congenital heart transplant: twenty-year
experience in a tertiary Brazilian hospital. Rev Bras Cir Cardiovasc.

[3] Jatene MB, Miana LA, Pessoa AJ, Riso A, Azeka E, Tanamati C (2008). Pediatric heart transplantation in refractory cardiogenic
shock: a critical analysis of feasibility, applicability and
results. Arq Bras Cardiol.
